# P-wave terminal force in lead V1 is associated with recurrence after catheter ablation in patients with paroxysmal atrial fibrillation and normal left atrial size

**DOI:** 10.3389/fcvm.2024.1467585

**Published:** 2024-10-10

**Authors:** Zhao Wang, Binhao Wang, Yiheng Yang, Xiaolei Yang, Ying Che, Yunlong Xia

**Affiliations:** ^1^ Department of Ultrasonography, The First Affiliated Hospital of Dalian Medical University, Dalian, Liaoning, China; ^2^ Department of Cardiology, The First Affiliated Hospital of Dalian Medical University, Dalian, Liaoning, China; ^3^ Department of Ultrasonography, The First Affiliated Hospital of Ningbo University, Ningbo, Zhejiang, China; ^4^ Arrhythmia Center, The First Affiliated Hospital of Ningbo University, Ningbo, Zhejiang, China

**Keywords:** P-wave, paroxysmal atrial fibrillation, radiofrequency catheter ablation, recurrence, electrical remodeling

## Abstract

**Background:**

A previous investigation reported that an abnormal P-wave terminal force in lead V1 (PTFV1) is a marker for electrical remodeling of the left atrium (LA). We aimed to assess the relationship of PTFV1 with LA tachyarrhythmia (LATA) recurrence after radiofrequency catheter ablation (RFCA) in patients with paroxysmal atrial fibrillation (PAF) and normal LA size.

**Methods:**

Patients with PAF and normal LA size (LA volume index < 34 ml/m^2^) who underwent RFCA were consecutively included between January 2018 and December 2020 and divided into two groups based on the presence (recurrence group) or absence (nonrecurrence group) of LATA recurrence. PTFV1 was measured according to preprocedural electrocardiography. The association between PTFV1 and the recurrence of LATA was investigated.

**Results:**

A total of 385 patients were included. After a median follow-up period of 745 (467, 977) days, 109 (28.3%) patients experienced LATA recurrence. PTFV1 was greater in the recurrence group. Multivariate Cox regression analysis demonstrated that the hazard ratio and 95% confidence interval for PTFV1 per 1,000 μV*ms increase and PTFV1 > 4,000 μV*ms were 1.22 (1.13–1.32, *p* < 0.001) and 2.32 (1.54–3.48, *p* < 0.001), respectively.

**Conclusion:**

PTFV1 is an independent predictor for LATA recurrence after RFCA in patients with PAF and normal LA size.

## Introduction

Atrial fibrillation (AF) is a common arrhythmia seen in clinical practice (1). Ischemic stroke and heart failure are serious complications of AF (2). Radiofrequency (RF) catheter ablation (RFCA) is an effective and safe therapy for AF with a success rate as high as 60%–90% (3–5). However, there is still a risk of recurrence for patients, with a rate of 30%–50% (6, 7). At present, many studies have found that age, left atrial (LA) enlargement (4), diabetes mellitus (8), obesity and sleep apnea (9), hypertrophic cardiomyopathy (10) and inflammatory factors (11) can affect the risk of recurrence after RFCA. These conditions have often been accompanied by changes in LA structure and function. It is important to timely and accurately identify the predictors of recurrence to choose a suitable candidate for RFCA. A recent study found that electrical remodeling of the LA is a better predictor for recurrence than structural remodeling in AF patients undergoing RFCA (12). P-wave terminal force in lead V1 (PTFV1) derived from electrocardiography (ECG) at sinus rhythm is a marker for atrial electrical dysfunction (13). However, the data regarding the relationship between PTFV1 and recurrence after RFCA are controversial and limited. This study aimed to investigate the association of PTFV1 with the recurrence of LA tachyarrhythmia (LATA) after RFCA in patients with paroxysmal AF (PAF) and normal LA size.

## Methods

### Study population

From January 2018 to December 2020, patients with symptomatic PAF who were admitted to the First Affiliated Hospital of Dalian Medical University for RFCA were consecutively screened. All clinical, echocardiography and blood test data were collected. The CHA_2_DS_2_-VASc score was calculated by two investigators for each patient based on comorbidities (14). The following inclusion criteria were met: (1) patients with nonvalvular PAF who underwent RFCA for the first time; (2) patients with ECG recording at sinus rhythm before RFCA; (3) patients whose body surface area-indexed LA volume (LAVI) was <34 ml/m^2^ as measured by preprocedural transthoracic echocardiography (TTE) (15); and (4) patients without an LA thrombus confirmed by preprocedural transesophageal echocardiography. The exclusion criteria were as follows: (1) patients with AF in the setting of moderate-to-severe mitral stenosis and/or in the presence of a mechanical heart valve; (2) patients with an LA thrombus; (3) patients with hyperthyroidism, a cardiac function grade of 4 (New York cardiac function class), severe hepato-renal insufficiency, severe chronic obstructive pulmonary disease, acute coronary syn-drome or stroke; and (4) patients with incomplete electrical isolation of the pulmonary vein (PV). This study was approved by the Ethics Committee of the First Affiliated Hospital of Dalian Medical University and complies with the Declaration of Helsinki (YJ-KY-2021-123). Informed consent was obtained from all study participants.

### Definition of PTFV1 and LA Size

The 12-lead ECG at sinus rhythm was recorded and analyzed in all patients on the day of the procedure. PTFV1 was defined as the absolute value of the depth (μV) times the duration (ms) of the downward deflection (terminal portion) of the P-wave in lead V1. PTFV1 was automatically measured using the GE Marquette 12-SL program (GE Marquette, Milwaukee, WI) as previously reported (16). The cutoff value for PTFV1 was set to 4,000 μV*ms according to previous studies (17, 18).

Preprocedural TTE was performed in all patients. LA diameter, LA volume (LAV), LAVI, and left ventricular ejection fraction (LVEF) were measured and recorded. According to the recommendations by the American Society of Echocardiography and the European Association of Cardiovascular Imaging (11), two-dimensional volumetric measurements were based on tracings of the blood-tissue interface on apical four- and two-chamber views. Atrial appendage and PVs were excluded from LAV measurement. Maximal LAV was defined as end-systolic LAV prior to the opening of the mitral valve. The LAVI was then calculated by adjusting for body surface area. Patients with LAVI < 34 ml/m^2^ were considered to have normal LA size.

### RFCA procedure

Antiarrhythmic drugs were stopped five half-lives before the procedure. Oral anticoagulants were continued uninterrupted periprocedurally. The RFCA procedure was performed under local anesthesia using dezocine for analgesia. Under fluoroscopic guidance, a multipolar catheter (MicroPort, Shanghai, China) was placed in the coronary sinus through the right femoral vein. Two transseptal sheaths (Synaptic Medical, Beijing, China) were introduced into the right femoral vein. After double transseptal punctures, anticoagulation was started by a bolus administration of 100 IU/kg heparin followed by continuous intravenous heparin infusion to maintain an activated clotting time of 300–350 s. An ablation catheter (SmartTouch, Biosense Webster, Diamond Bar, USA) and a multispline (PentaRay, Biosense Webster, Diamond Bar, USA) or circular (MicroPort, Shanghai, China) mapping catheter were advanced to the LA through the two transseptal sheaths. Electroanatomic three-dimensional mapping of the LA and PV was performed by the Carto3 system (Biosense Webster, Diamond Bar, USA). The saline irrigation flow was 2 ml/min during catheter manipulation and 17 ml/min during RFCA. RF delivery was performed at a constant power of 35 W or 30 W (when at posterior left inferior PV). PV isolation (PVI) was performed in all patients by point-to-point RFCA. If a trigger originated from the superior vena cava (SVC) after bi-lateral PVI, segmental isolation of the SVC was needed. After PVI and SVC isolation (if necessary), 30 min was taken for monitoring the bidirectional block. If conduction recovery occurred, reablation was performed for isolation.

### Follow-up

All patients received antiarrhythmic drug therapy and anticoagulants for 3 months. Twenty-four-hour Holter recordings were scheduled at 3, 6, 9, and 12 months and every 6 months thereafter. Patients with self-reported symptoms of palpitation and chest tightness were immediately sent to the hospital for a 12-lead ECG and/or 24-h Holter. The recurrence of LATA was defined as documented atrial tachycardia, atrial flutter or AF episodes lasting more than 30 s after a 3-month blanking period following RFCA. Recurrence of tachyarrhythmia originating from the right atrium was excluded.

### Statistical analysis

Patients were divided into two groups based on the presence (recurrence group) or absence (nonrecurrence group) of LATA recurrence during follow-up. Comparisons of characteristics between patients with PTFV1 > 4,000 μV*ms and those with PTFV1 ≤ 4,000 μV*ms were also performed. Normally distributed continuous variables are expressed as the mean (standard deviation), while the median (interquartile range) is used for variables with a skewed distribution. Categorical variables are expressed as absolute numbers (percentages). Continuous variables were compared using the t test and Mann–Whitney *U* test for normally and nonnormally distributed data, respectively. Categorical variables were compared using the chi-square test or Fisher's exact test where appropriate.

Survivor functions were estimated for AF recurrence in each group using the Kaplan–Meier method and statistically evaluated using a log-rank test for trend. We used the Cox proportional hazards model to calculate adjusted hazard ratios (HRs) and corresponding 95% confidence intervals (CIs) of PTFV1 for predicting the recurrence of AF. PTFV1 was modeled as a continuous variable (per 1,000 μV*ms increase) or a categorical variable (using PTFV1 ≤ 4,000 μV*ms as a reference). The variables with statistical significance (*p* < 0.05) in the univariate analysis were included in the multivariate Cox regression model. Statistical analyses were performed with SPSS 19.0 (IBM, Armonk, NY, USA), and *p* < 0.05 (2-tailed) was considered statistically significant.

## Results

### Comparison of clinical characteristics between patients with or without LATA recurrence

**Figure 1:**
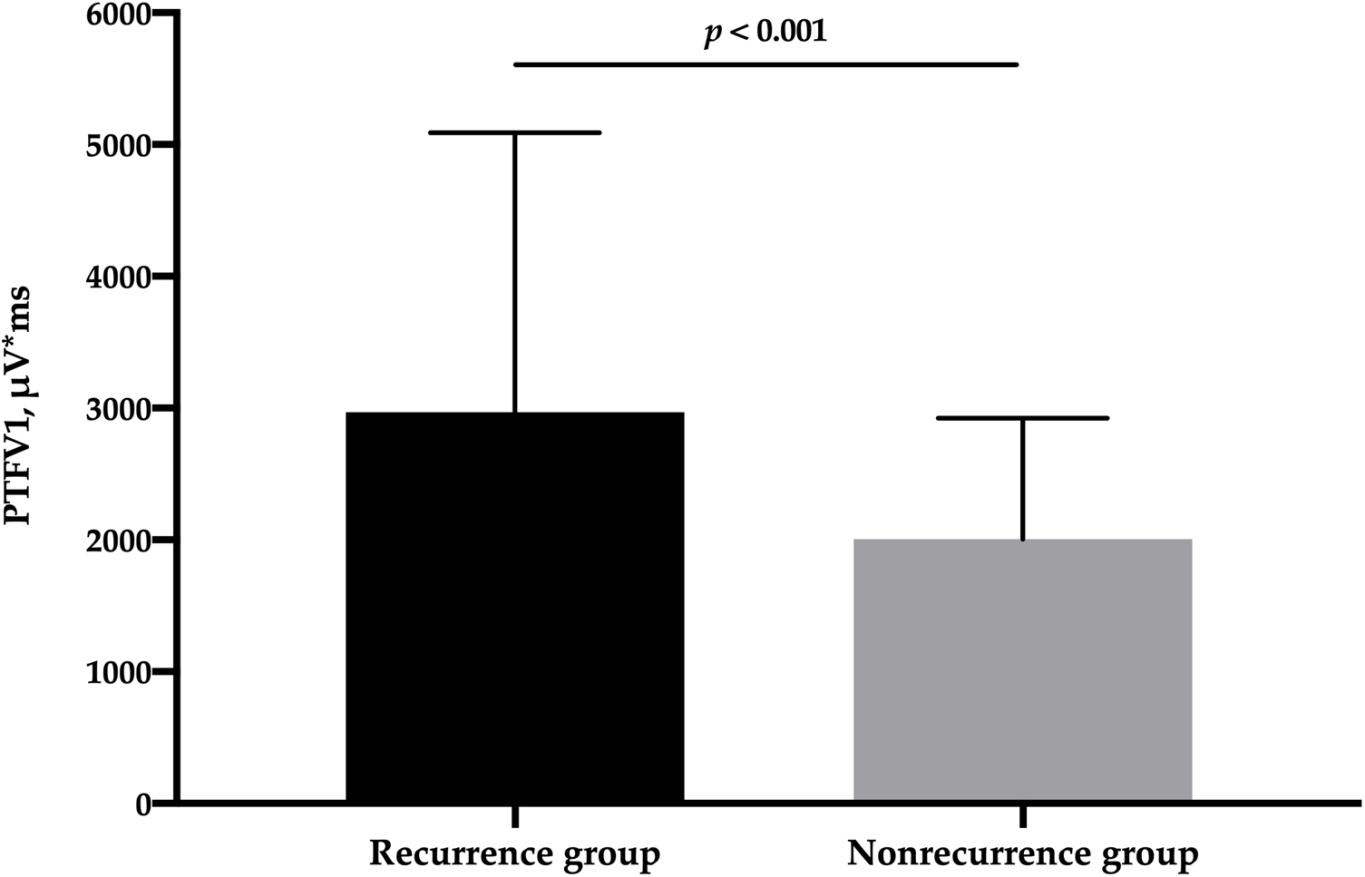
Comparison of PTFV1 between patients with and without LATA recurrence. LATA, left atrial tachyarrhythmia; PTFV1, P-wave terminal force in lead V1.

A total of 385 patients with PAF and normal LA size were included in this study. Complete PVI was achieved in all patients. During a median follow-up period of 745 (467, 977) days, 109 (28.3%) patients experienced LATA recurrence (AF, *n* = 98; atrial flutter, *n* = 7; atrial tachycardia, *n* = 4). PTFV1 in the recurrence group was significantly higher than that in the nonrecurrence group [2,968 (2,143, 5,090) μV*ms vs. 2,006 (1,416, 2,924) μV*ms, *p* < 0.001] (Figure 1). The percentage of PTFV1 > 4,000 μV*ms in the recurrence group was significantly higher than that in the nonrecurrence group (36.7% vs. 12.7%, *p* < 0.001). In addition, P-wave duration was higher in the recurrence group (114.9 ± 12.9 ms vs. 110.4 ± 14.6, *p* = 0.005). However, LA diameter, LAV, and LAVI were comparable between the two groups (Table 1).

**Table 1:** Comparison of clinical characteristics between patients with or without LATA recurrence.

Variable	Recurrence group	Nonrecurrence group	*p* value
*n*	109	276	
Age, years	63.6 ± 9.5	64.7 ± 9.9	0.330
Male, *n* (%)	56 (51.4)	162 (58.7)	0.192
Body mass index, kg/m^2^	23.9 ± 2.4	24.5 ± 3.4	0.056
Time between the first AF episode and ablation, months	12 (3, 36)	12 (5, 24)	0.248
Congestive heart failure, *n* (%)	24 (22.0)	65 (23.6)	0.748
Hypertension, *n* (%)	50 (45.9)	133 (48.2)	0.682
Diabetes mellitus, *n* (%)	14 (12.8)	41 (14.9)	0.611
Coronary artery disease, *n* (%)	7 (6.4)	13 (4.7)	0.610
CHA_2_DS_2_-VASc score	1.7 ± 1.4	1.8 ± 1.4	0.338
LA diameter, mm	37.6 ± 2.9	37.4 ± 3.3	0.494
LAV, ml	43.5 ± 8.6	42.6 ± 9.9	0.391
LAVI, ml/m^2^	23.0 ± 4.4	22.5 ± 4.8	0.367
LVEF,%	58.1 ± 2.3	58.0 ± 2.7	0.870
P-wave duration, ms	114.9 ± 12.9	110.4 ± 14.6	0.005
PTFV1, μV*ms	2,968 (2,143, 5,090)	2,006 (1,416, 2,924)	<0.001
PTFV1 > 4,000 μV*ms, *n* (%)	40 (36.7)	35 (12.7)	<0.001
eGFR, ml/min/1.73 m^2^	94.7 ± 11.7	91.6 ± 15.3	0.035
Uric acid, μmol/Ll	343.8 ± 88.6	333.1 ± 74.9	0.269
Antiarrhythmic therapy before ablation			0.971
Amiodarone	10 (9.2)	26 (9.4)	
Propafenone	16 (14.7)	39 (14.1)	
β-blocker	31 (28.4)	85 (30.8)	
Antiarrhythmic therapy after ablation			0.066
Amiodarone	45 (41.3)	120 (43.5)	
Propafenone	50 (45.9)	140 (50.7)	
β-blocker	14 (12.8)	16 (5.8)	

eGFR, estimated glomerular filtration rate; LA, left atrium; LATA, LA tachyarrhythmia; LAV, LA volume; LAVI, body surface area-indexed LAV; PTFV1, P-wave terminal force in lead V1.

### Comparison of clinical characteristics between patients with or without abnormal PTFV1

There were 75 patients with PTFV1 > 4,000 μV*ms and 310 with PTFV1 ≤ 4,000 μV*ms. The LATA recurrence rate in patients with PTFV1 > 4,000 μV*ms was significantly higher than that in those with PTFV1 ≤ 4,000 μV*ms (53.3% vs. 22.3%, *p* < 0.001). The P-wave duration (116.6 ± 10.9 ms vs. 110.5 ± 14.8, *p* < 0.001), LAV (46.6 ± 8.0 ml vs. 41.9 ± 9.8, *p* < 0.001) and LAVI (24.8 ± 4.3 vs. 22.1 ± 4.7, *p* < 0.001) were greater in patients with PTFV1 > 4,000 μV*ms (Table 2).

**Table 2:** Comparison of clinical characteristics between patients with or without abnormal PTFV1.

Variable	>4,000 μV*ms	≤4,000 μV*ms	*p* value
*n*	75	310	
Age, years	66.1 ± 9.0	63.9 ± 9.9	0.087
Male, *n* (%)	45 (60.0)	173 (55.8)	0.511
Body mass index, kg/m^2^	23.8 ± 2.5	24.5 ± 3.3	0.065
Time between the first AF episode and ablation, months	12 (3, 36)	12 (4, 24)	0.740
Congestive heart failure, *n* (%)	19 (25.3)	70 (22.6)	0.612
Hypertension, *n* (%)	41 (54.7)	142 (45.8)	0.168
Diabetes mellitus, *n* (%)	14 (18.7)	41 (13.2)	0.227
Coronary artery disease, *n* (%)	4 (5.3)	16 (5.2)	1.000
CHA_2_DS_2_-VASc score	2.0 ± 1.4	1.7 ± 1.3	0.052
LA diameter, mm	38.6 ± 3.3	37.2 ± 3.1	<0.001
LAV, ml	46.6 ± 8.0	41.9 ± 9.8	<0.001
LAVI, ml/m^2^	24.8 ± 4.3	22.1 ± 4.7	<0.001
LVEF,%	57.6 ± 3.1	58.1 ± 2.4	0.152
P-wave duration, ms	116.6 ± 10.9	110.5 ± 14.8	<0.001
PTFV1, μV*ms	5,088 (4,466, 5,976)	1,982 (1,410, 2,575)	<0.001
eGFR, ml/min/1.73 m^2^	90.7 ± 14.7	92.9 ± 14.4	0.251
Uric acid, μmol/L	337.4 ± 83.4	335.9 ± 78.1	0.879
Antiarrhythmic drugs before ablation			0.666
Amiodarone	6 (8.0)	30 (9.7)	
Propafenone	11 (14.7)	44 (14.2)	
β-blocker	19 (25.3)	97 (31.3)	
Antiarrhythmic drugs after ablation			0.730
Amiodarone	30 (40.0)	135 (43.5)	
Propafenone	40 (53.3)	150 (48.4)	
β-blockers	5 (6.7)	25 (8.1)	
LATA recurrence, *n* (%)	40 (53.3)	69 (22.3)	<0.001

AF, atrial fibrillation; eGFR, estimated glomerular filtration rate; LA, left atrium; LATA, LA tachyarrhythmia; LAV, LA volume; LAVI, body surface area-indexed LAV; PTFV1, P-wave terminal force in lead V1.

### The value of PTFV1 in predicting LATA recurrence

**Figure 2:**
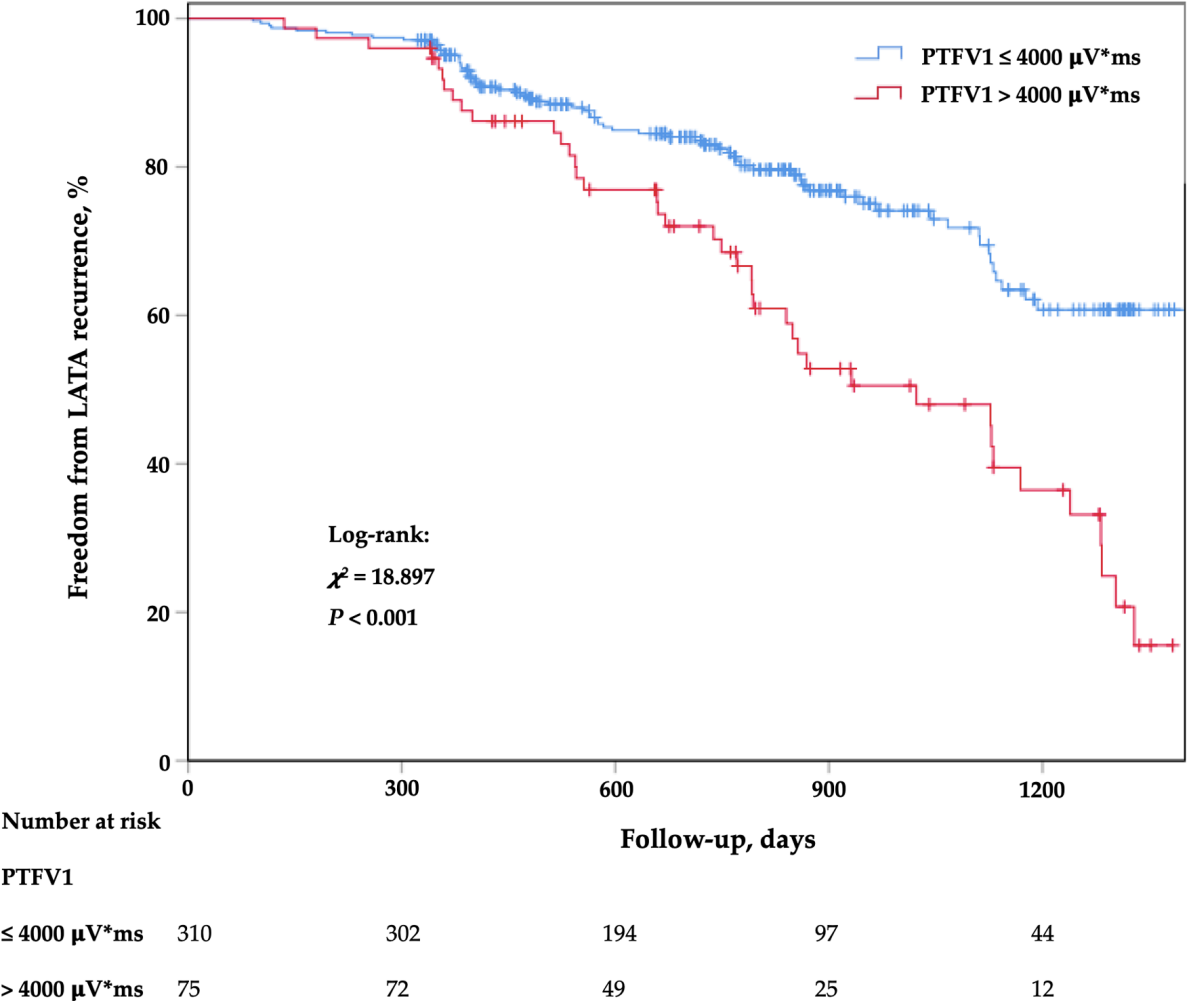
Kaplan–meier cumulative event-free curves of LATA. LATA, left atrial tachyarrhythmia; PTFV1, P-wave terminal force in lead V1.

The Kaplan–Meier survival curve showed that the LATA recurrence rate was higher in patients with PTFV1 > 4,000 μV*ms (Figure 2). After adjusting for all variables potentially associated with recurrence, PTFV1 (per 1,000 μV*ms increase) was the only independent predictor of LATA recurrence (HR = 1.22, 95% CI 1.13–1.32, *p* < 0.001). The risk of LATA recurrence in patients with PTFV1 > 4,000 μV*ms was 2.32 times that in those with PTFV1 ≤ 4,000 μV*ms (HR = 2.32, 95% CI 1.54–3.48, *p* < 0.001). In addition, LAVI was not related to LATA recurrence (Table 3).

**Table 3:** Univariate and multivariate Cox regression models to identify the predictors for LATA recurrence.

	Univariate analysis		Multivariate analysis				
	HR (95% CI)	*p* value	Model 1			Model 2	
			HR (95% CI)	*p* value		HR (95% CI)	*p* value
Age, years	0.99 (0.97–1.01)	0.182	0.98 (0.95–1.01)	0.066		0.98 (0.96–1.01)	0.121
Male	0.75 (0.51–1.09)	0.133	0.65 (0.44–0.96)	0.082		0.71 (0.48–1.05)	0.086
CHA_2_DS_2_-VASc score	1.01 (0.88–1.16)	0.884	1.11 (0.94–1.32)	0.223		1.09 (0.92–1.29)	0.328
eGFR, ml/min/1.73 m^2^	1.01 (0.99–1.03)	0.236	1.01 (0.99–1.03)	0.413		1.01 (0.99–1.03)	0.420
LAVI, ml/m^2^	1.00 (0.07–1.05)	0.830	0.98 (0.93–1.02)	0.294		0.98 (0.94–1.02)	0.303
P-wave duration, ms	1.02 (1.00–1.03)	0.018	1.01 (0.99–1.03)	0.089		1.02 (1.00–1.03)	0.055
PTFV1, per 1,000 μV*ms	1.20 (1.11–1.28)	<0.001	1.22 (1.13–1.32)	<0.001		-	-
PTFV1 > 4,000 μV*ms	2.32 (1.57–3.42)	<0.001	-	-		2.32 (1.54–3.48)	<0.001

eGFR, estimated glomerular filtration rate; LATA, LA tachyarrhythmia; LAVI, body surface area-indexed left atrial volume; PTFV1, P-wave terminal force in lead V1.

## Discussion

### Main findings

To the best of our knowledge, this is the first investigation regarding the association of PTFV1 with LATA recurrence after RFCA in patients with PAF and normal LA size. The results showed that PTFV1 was an independent predictor for LATA recurrence after RFCA. However, LAVI was not associated with recurrent LATA in such patients.

### Electrical remodeling and structural remodeling in AF

LA remodeling progresses in a series of electrical remodeling, subsequent contractile remodeling, and finally structural remodeling (19). LA enlargement is a consequence of structural remodeling due to AF. Many studies have found that LA enlargement is the main factor affecting success and recurrence after RFCA (20–22). However, other investigations demonstrated that LA size failed to predict recurrence after RFCA in patients with PAF (23, 24). In the present study, LA size was also not related to the recurrence rate after RFCA. According to previous investigations, the majority of patients with PAF had a normal LA size. Arroja et al. reported that patients with PAF without overt structural heart disease have electrical remodeling (25). Watanabe et al. found that a low-voltage zone (LVZ) exists in the early remodeling phase in patients with PAF whose LA has not been dilated (26). Kim et al. found that electrical remodeling of the LA is a better predictor for recurrence than structural remodeling in AF patients undergoing RFCA (12). Therefore, LA electrical modeling may play a vital role in the maintenance of AF and recurrence after RFCA in patients with PAF and normal LA size.

### PTFV1 and AF

Previous research indicated that LA electrical remodeling in AF was better represented by LVZs (27). However, LVZ is an invasive parameter obtained by voltage mapping during RFCA. Therefore, it is better to develop simple and noninvasive tools for physicians to evaluate LA electrical remodeling before RFCA. Indices from P-waves, such as P-wave duration and P-wave amplitude, were used to predict recurrence after RFCA in patients with AF. Park et al. found that a low P-wave amplitude (<0.1 mV) in lead I was related to LA remodeling and independently predicted clinical recurrence after RFCA in patients with PAF (HR = 2.16, 95% CI 1.31–3.58, *p* = 0.003) (23). A meta-analysis indicated that P-wave duration with a cutoff of >120 ms to >150 ms at sinus rhythm before RFCA may be associated with recurrence after PVI (28). Amplified P-wave duration has been associated with LVZs (29). It can predict new-onset AF in patients with heart failure with preserved ejection fraction (30). Further investigation has reported that amplified P-wave duration can predict recurrence after cryoballoon ablation in patients with persistent and long-standing persistent AF (31). However, the anterior initial vector of the P-wave in ECG is right atrial activation, and the posterior terminal vector is left atrial activation; hence, it is called the left and right atrial comprehensive depolarization wave. Therefore, parameters derived from the latter part of the P-wave may be more suitable to represent LA remodeling. PTFV1 represents the area of the transverse left atrial depolarization vector, and the increase in PTFV1 represents a larger left atrial depolarization vector, indicating abnormal left atrial interatrial conduction.

PTFV1 is now used as a tool to evaluate patients for atrial cardiomyopathy and for the risk of AF (13, 18, 32). A large cohort study found that PTFV1 is an independent risk factor for new-onset AF in the normal population (33). Li et al. found that increasing PTFV1 was associated with new-onset AF in patients experiencing acute myocardial infarction (18). Goda et al. reported that PTFV1 was clearly related to new-onset AF in patients with unexplained stroke (34).

According to the study by Martín et al., PTFV1 was a predictor for recurrent AF after cardioversion (35). However, the data regarding the relationship between PTFV1 and recurrence after RFCA in patients with AF are controversial and limited. Park et al. reported that abnormal PTFV1 was not associated with clinical recurrence after RFCA in patients with PAF (23). Similar result was found in the study of Qiu et al. (36). We have to point out that the mean values of LAVI and LA diameter measured by echocardiography were 30.3 ± 9.6 ml/m^2^ and 39.4 ± 5.3 mm respectively in their studies, indicating that quite a few patients had LA enlargement. LA enlargement (structural remodeling) may weaken the utility of abnormal PTFV1 (electrical remodeling) in predicting recurrence after RFCA. In the research of Li et al., the AF recurrence rate was significantly higher in patients with PTFV1 > 4,000 μV*ms after RFCA (log-rank test: χ*^2^* = 4.739, *p* < 0.001) (17). Sudo et al. reported that the PTFV1 at 3 months after AF ablation could be a valuable noninvasive predictor of recurrence in patients with persistent AF (HR = 2.12, 95% CI 1.44–3.13, *P* < 0.001) (37). In the present study, we found that either an increasing PTFV1 (per 1,000 μV*ms increase) or an abnormal PTFV1 (>4,000 μV*ms) was an independent predictor for LATA recurrence after RFCA in patients with PAF and normal LA size. Therefore, PTFV1 may be valuable for use as a new noninvasive ECG index to evaluate the recurrence of LATA after RFCA during clinical practice.

### Clinical implications

PTFV1 is a strong predictor of LA electrical remodeling. Previous study demonstrated that PTFV1 was independently associated with the of LVZs in patients with PAF (36). For patients with PAF and abnormal PTFV1, voltage mapping and LVZs modification may be necessary to improve the outcomes of patients undergoing RFCA. As mentioned above, PTFV1 has been proved to be a predictor of recurrence after RFCA. Patients with abnormal PTFV1 before procedure may need careful medical treatment after ablation, such as the continuation of anticoagulation and antiarrhythmic drugs, as well as intensive clinical follow-up.

### Study limitations

This study may have several limitations and they are as follows. First, this was a single-center retrospective study, and the results should be confirmed by further multicenter prospective investigations. Second, only patients with PAF and normal LA size were included. Whether the findings could be extended to patients with LA enlargement or patients with persistent, long-standing persistent or permanent AF is unknown. Third, voltage mapping data were not available for quite a few patients; therefore, the relationship between PTFV1 and LVZs cannot be analyzed. Finally, 24-h Holter was used to detect the LATA recurrence and AF burden was not available. Therefore, some recurrences may be missed.

## Conclusion

PTFV1 derived from preprocedural ECG is an independent predictor for LATA recurrence after RFCA in patients with PAF and normal LA size.

## Data Availability

The original contributions presented in the study are included in the article/Supplementary Material, further inquiries can be directed to the corresponding authors.
